# OA07.02. Naturopathic medicine for the prevention of cardiovascular disease: a pragmatic randomized clinical trial

**DOI:** 10.1186/1472-6882-12-S1-O26

**Published:** 2012-06-12

**Authors:** D Seely, O Szczurko, C Kieran, H Fritz, P Herman, R Bradley, S Aberdour, C Herrington, P Rouchotas, D Lescheid, T Gignac, B Bernhardt, Q Zhou, G Guyatt

**Affiliations:** 1 Ottawa Integrative Cancer Centre, Ottawa, Canada; 2 Canadian College of Naturopathic Medicine, Toronto, Canada; 3 University of Arizona, Tucson, USA; 4 Bastyr University, Kenmore, USA; 5 Private clinic, Vancouver, Canada; 6 Private Clinic, Halifax, Canada; 7 McMaster University, Hamilton, Canada

## Purpose

Cardiovascular disease (CVD) is largely preventable through a number of dietary and lifestyle based interventions utilized in naturopathic medicine. We aimed to test the whole-practice of this discipline for the prevention of cardiovascular disease in a work place setting.

## Methods

Multi-site pragmatic randomized controlled trial of enhanced usual care (EUC; usual care plus biometric screening) versus EUC plus naturopathic care (EUC+NC). NC consisted of individualized care provided in work-site clinics by licensed naturopathic doctors (NDs) utilizing one or more of the following strategies: lifestyle counseling, nutritional medicine, and/or dietary supplementation. EUC consisted of usual care provided by the participant's family physician in the community following identification as having a higher relative risk of developing cardiovascular disease. Primary outcomes were incidence of metabolic syndrome and 10-year risk of having a cardiovascular event based on the Framingham algorithm and the Adult Treatment Panel (ATP) III diagnostic criteria for metabolic syndrome.

## Results

A total of 246 participants were randomized and enrolled in study work-sites in three cities across Canada; 207 participants completed the study. The two groups were similar at baseline. After one year of individualized naturopathic care, there was a 3.6 percentage point reduction in 10-year cardiovascular risk (95% CI: -5.1, -2.3) and a 27.4% reduced prevalence of metabolic syndrome (95% CI; -41.7, -13.1) in the treatment arm (n=124) compared to the control arm (n=122).

## Conclusion

This study is the first pragmatic whole-practice trial to formally evaluate the benefits of individualized naturopathic care for the prevention of cardiovascular disease. In this setting, and widely generalizable due to the whole-systems methodology employed, naturopathic medicine appears to provide safe and effective risk reduction for people at risk of developing cardiovascular disease.

